# LINKING LIVES: PROMOTING HEALTH EQUITY TO DISRUPT THE CYCLE OF SOCIAL ISOLATION

**DOI:** 10.1093/geroni/igz038.723

**Published:** 2019-11-08

**Authors:** Karen Fredriksen Goldsen

**Affiliations:** University of Washington, Seattle, Washington, United States

## Abstract

Resulting from social, economic, and environmental disadvantage in vulnerable communities, health inequities have systematically created greater obstacles to aging. Despite the adversity of their lives, or perhaps because of it, older adults from vulnerable populations embody an Iridescent Life Course, displaying remarkable resilience and resistance, yet are at greater risk of health disparities and social isolation in later life. In the U.S. social isolation has reached epidemic proportions, creating a largely invisible public health crisis with few resources for an aging population. In her presentation, Professor Karen Fredriksen Goldsen will share her landmark longitudinal research on LGBT older adults, who have historically confronted social exclusion, with divergent pathways leading to social isolation. She will discuss the development and testing of community evidence-based interventions and how these findings can provide effective alternatives for anyone living in isolation as well as promote health equity, creating a future for full aging potential.

